# High-risk clonal expansion of IMP-producing *Enterobacter cloacae* complex in regional Japanese healthcare settings

**DOI:** 10.1128/aac.00953-25

**Published:** 2025-10-17

**Authors:** Kentarou Takei, Hajime Kanamori, Ryuji Sakata, Miho Ogawa, Hiroaki Baba, Yumiko Takei, Asami Nakayama, Tetsuji Aoyagi

**Affiliations:** 1 Department of Infectious Diseases, Internal Medicine, Tohoku University Graduate School of Medicine 38047, Sendai, Japan; 2 Department of Infectious Diseases and Laboratory Medicine, Kanazawa University 12858, Kanazawa, Japan; 3 Department of Bacteriology, BML Inc., Kawagoe, Japan; Shionogi Inc., Florham Park, New Jersey, USA

**Keywords:** carbapenemase-producing enterobacterales, IMP-1, high-risk clones, enterobacter cloacae complex

## Abstract

Carbapenemase-producing *Enterobacter cloacae* complex (CPEC) represents an emerging threat in healthcare settings; however, its spread across local and regional hospitals remains elusive. Between 2014 and 2022, 211 non-duplicate *E. cloacae* complex isolates (ECC) with reduced carbapenem susceptibility based on automated susceptibility testing were collected from 58 hospitals in 14 prefectures across Japan. PCR detected the *bla*_IMP-1_ group in 92.9% of isolates. Among these, 80 representative CPEC isolates underwent whole-genome sequencing. Two dominant high-risk clonal groups, ST78 and ST133 (including single-locus variants), accounted for 62.5% of sequenced isolates. Notably, ST133—rarely reported in Japan—was the second most common type and detected widely, suggesting gradual and inconspicuous dissemination. ST78 exhibited substantial genomic diversity, frequent co-carriage of extended-spectrum beta-lactamase genes, and multiple plasmid replicons, while ST133 formed a more uniform lineage with limited resistance determinants. Most isolates remained highly susceptible to amikacin and cefiderocol. Core-genome multi-locus sequence typing suggested possible intra-facility persistence and inter-facility transmission of closely related strains (≤10 allele differences) in some hospitals over time. Among the 46 facilities with *bla*_IMP-1_ group-positive isolates, 73.9% (34/46) were small hospitals (<200 beds). The proportion among screened ECC isolates was similarly high in small and large hospitals (90.5% vs 94.8%), with no significant difference, suggesting the need to extend molecular surveillance even in resource-limited settings. These findings highlight the utility of molecular confirmation and targeted genomic analysis, particularly in regions or settings where high-risk CPEC clones may circulate undetected, to support timely detection and containment efforts.

## INTRODUCTION

*Enterobacter cloacae* complex (ECC) comprises gram-negative, facultative anaerobic bacteria prevalent in healthcare settings and recognized as notable opportunistic pathogens (1). ECC commonly causes nosocomial infections—bloodstream, respiratory, urinary tract, and surgical site infections (2). Although *Klebsiella pneumoniae* predominates among carbapenem-resistant *Enterobacterales* (CRE) worldwide (3), ECC has recently become a major CRE contributor owing to its intrinsic resistance and ability to acquire carbapenemase genes via mobile elements (4). While *Klebsiella pneumoniae* carbapenemase (KPC), New Delhi metallo-β-lactamase (NDM), and OXA-48-like enzymes are predominant in other parts of the world (5), Japan is characterized by the widespread presence of imipenemase (IMP)-type metallo-β-lactamases, accounting for approximately 25% of detections worldwide (6). In Japan, *bla*_IMP-1_ is the most prevalent, while *bla*_IMP-6_ is also common in certain areas (6–8). IMP-producing ECC represents the principal carbapenemase threat in Japan, and this restricts treatment options and worsens clinical outcomes (9). Although nationwide CRE surveillance has been conducted since 2014 (10), molecular epidemiology—especially of carbapenemase-producing *E. cloacae* complex (CPEC)—has focused on tertiary and university hospitals (11–13), institutions at elevated risk for CRE introduction and transmission due to their central role in patient transfer networks (14, 15).

Globally, community hospitals and long-term acute care hospitals are known reservoirs for prolonged colonization and silent transmission of carbapenemase-producing *Enterobacterales* (CPE) (16), yet molecular data from such settings in Japan remain scarce. Mathematical modeling has shown that CRE transmission is shaped by patient movement and inter-facility networks—including non-tertiary hospitals—highlighting their critical role in regional dissemination (17, 18).

To address this gap, we conducted a regionally stratified genomic analysis of ECC isolates from local and regional hospitals across Japan to characterize CPEC clonal lineages and resistance profiles and to inform targeted infection-control strategies.

## RESULTS

### Characteristics of collected ECC isolates

**Fig 1:**
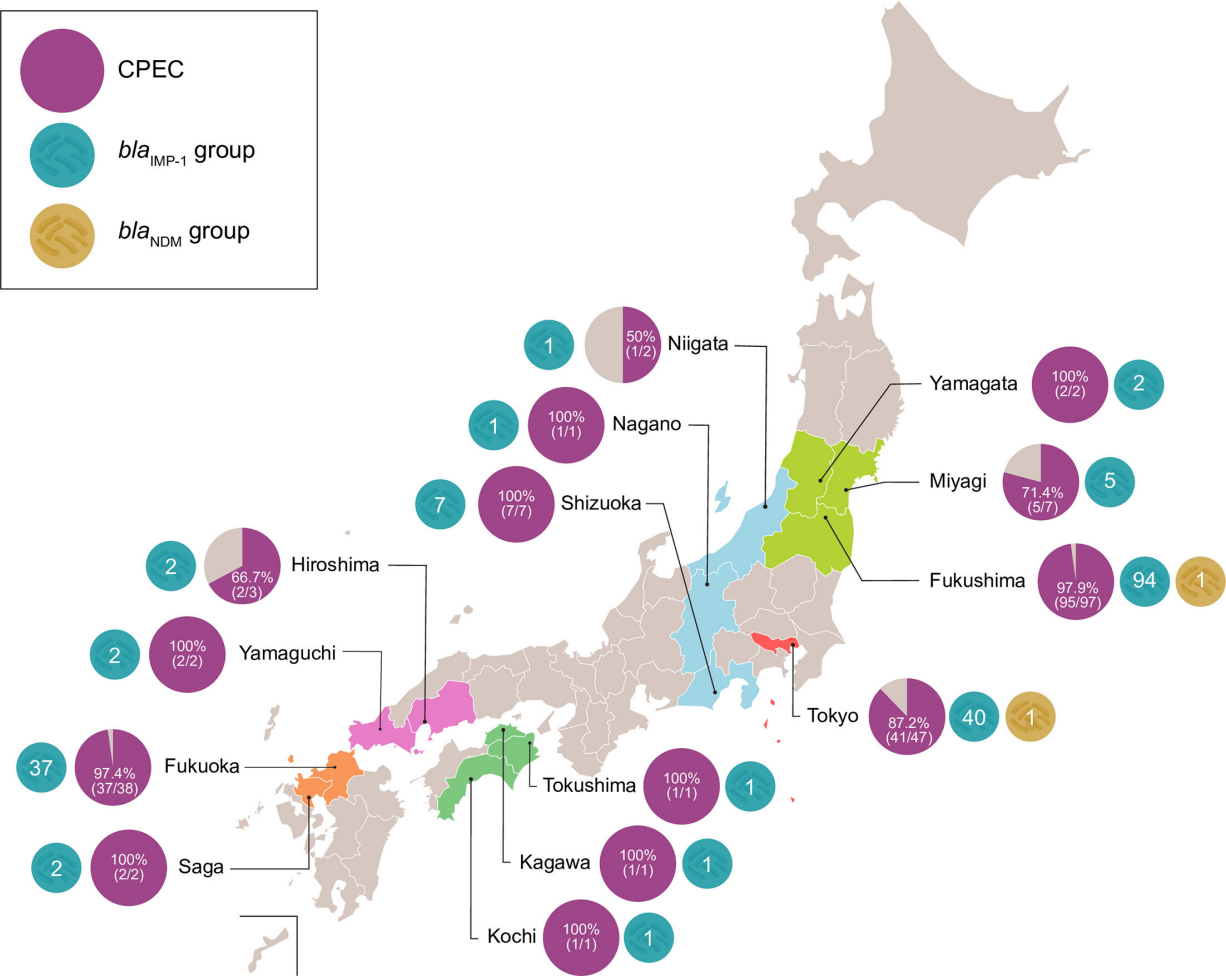
Regional distribution of the *bla*_IMP-1_ group in carbapenem-screening-positive *E. cloacae* complex. This map shows ECC isolates from 14 prefectures across Japan with reduced carbapenem susceptibility based on initial automated testing. Each pie chart represents all carbapenem-nonsusceptible isolates in a given prefecture (entire circle). The purple segment indicates isolates carrying any carbapenemase gene, with subcolors showing specific types: teal for *bla*_IMP-1_ group and yellow for *bla*_NDM_ group. Prefectures are color-coded by region as follows: light green (Tohoku), blue (Chubu), red (Tokyo), pink (Chugoku), green (Shikoku), and orange (Kyushu).

A total of 211 non‐duplicate ECC isolates flagged by an automated susceptibility testing system (Japan’s CRE criteria: meropenem minimum inhibitory concentration ≥2 µg/mL or imipenem MIC ≥2 µg/mL plus cefmetazole MIC ≥64 µg/mL) (10) were collected—one per patient (blood cultures prioritized)—from 58 local or regional hospitals in 14 prefectures (excluding university, psychiatric, and long‐term care facilities). Among the 211 screened ECC isolates, the most common specimen type was lower respiratory tract (79 isolates, 37.4%), followed by urine (56 isolates, 26.5%), upper respiratory tract (24 isolates, 11.4%), blood (23 isolates, 10.9%), and catheter-tip samples (eight isolates, 3.8%). The remaining 21 isolates (10.0%) were obtained from other specimen types, including abscesses, ascitic fluid, stool, bile, and pleural fluid. To evaluate all 211 ECC isolates for the presence of carbapenemase genes, we screened every isolate using PCR; overall, 196/211 (92.9%) were positive for the *bla*_IMP-1_ group (Fig. 1). Other patient demographics and specimen types are provided in the Supplemental material.

### Whole-genome sequencing and multi-locus sequence typing analysis: species identification, sequence types, and molecular profiles

**Fig 2:**
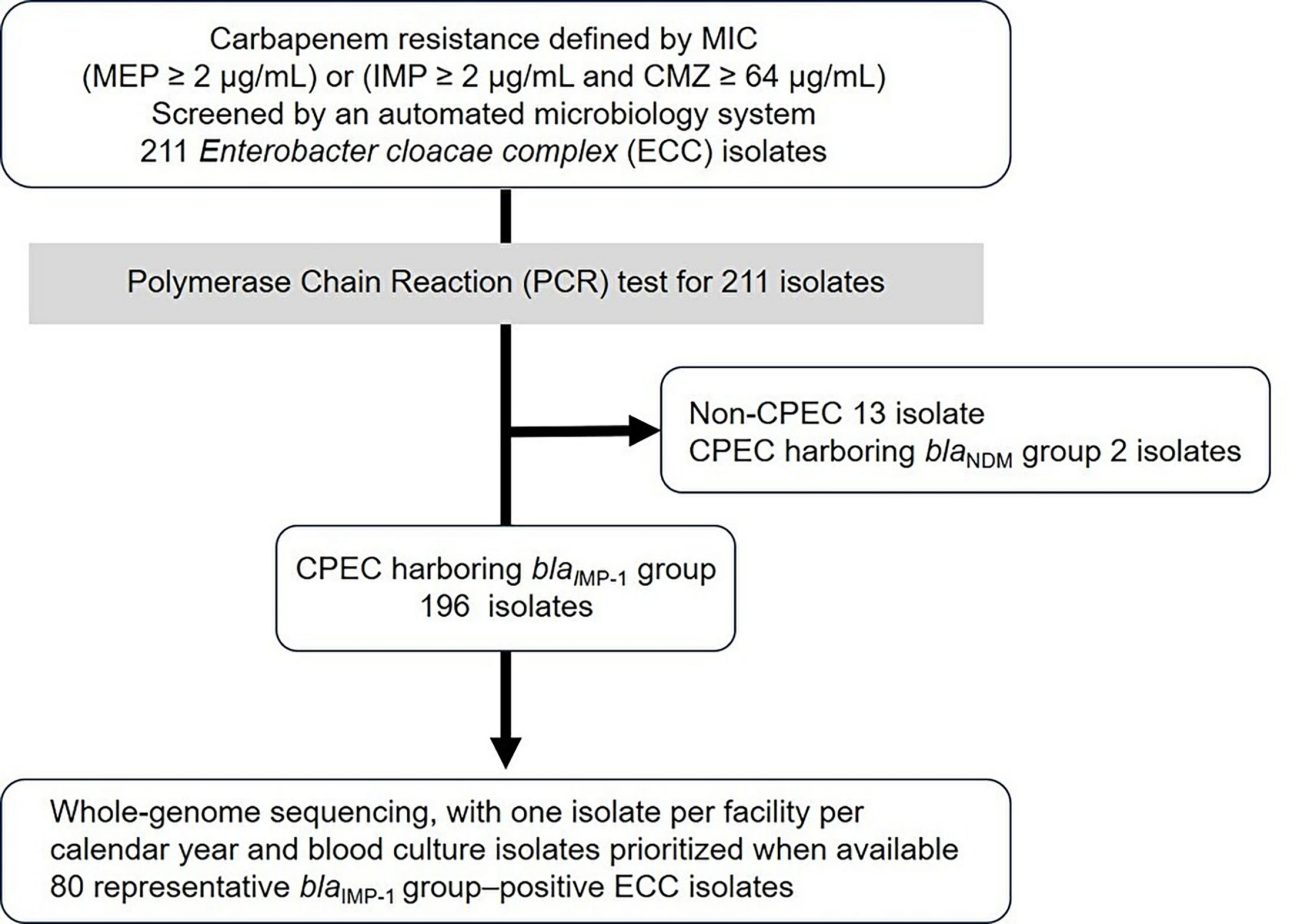
Study workflow for ECC isolate selection and analysis. This flowchart summarizes the screening, classification, and selection process for WGS. ECC isolates were collected based on Japan’s national CRE screening criteria using an automated microbiology system. All isolates underwent PCR testing for carbapenemase genes. One representative *bla*_IMP-1_ group-positive isolate per facility per calendar year was selected for sequencing, with preference given to blood culture isolates.

Among the 196 isolates confirmed as *bla*_IMP-1_ group-positive by PCR, one representative per facility per year (*n* = 80) underwent whole-genome sequencing (WGS) (Fig. 2). Draft assemblies had a mean coverage of 128× (range, 18–313×), a median of 108 contigs (range, 43–286), a median N50 of 253,091 bp (range, 116,829–466,726 bp), a mean genome size of 5,162,981 bp (range, 4.6–5.5 Mbp), a mean guanine-cytosine content of 54.9% (range, 54.3%–55.8%), and a mean of 4,907 coding sequences per isolate (range, 4,306–5,245). Detailed genome characteristics of all isolates are provided in Supplemental Table S2.

Species assignment by average nucleotide identity (ANI ≥95%) revealed a diverse array of *Enterobacter* species, most frequently *Enterobacter xiangfangensis* and *Enterobacter hoffmannii*. Furthermore, multi-locus sequence typing (MLST) identified ST78 (*n* = 25) and ST133 (*n* = 21) as the predominant types; including their single-locus variants yielded extended ST78 (*n* = 27) and ST133 (*n* = 23) groups, together comprising 50/80 (62.5%) of isolates (Table 1). Other detected species included *Enterobacter asburiae*, *Enterobacter bugandensis*, *Enterobacter chengduensis*, and unclassified *Enterobacter* spp.

**TABLE 1:** Distribution of sequence types and species identifications among CPEC isolates

ST group	ST	Species identification^*a*^	Number of strains, *n*
ST78 group	78	*Enterobacter hormaechei*	25
	3244^*b*^	*Enterobacter hormaechei*	1
	3323^*b*^	*Enterobacter hormaechei*	1
ST133 group	133	*Enterobacter xiangfangensis*	21
	3243^*b*^	*Enterobacter xiangfangensis*	2
Other STs group	175	*Enterobacter xiangfangensis*	5
	62	*Enterobacter xiangfangensis*	3
	93	*Enterobacter xiangfangensis*	3
	252	*Enterobacter asburiae*	3
	113	*Enterobacter xiangfangensis*	2
	742	*Enterobacter xiangfangensis*	2
	32	*Enterobacter bugandensis*	1
	32	*Enterobacter* sp.	1
	24	*Enterobacter asburiae*	1
	50	*Enterobacter xiangfangensis*	1
	66	*Enterobacter xiangfangensis*	1
	116	*Enterobacter xiangfangensis*	1
	118	*Enterobacter hormaechei*	1
	143	*Enterobacter xiangfangensis*	1
	414	*Enterobacter chengduensis*	1
	770	*Enterobacter* sp.	1
	3031^*b*^	*Enterobacter asburiae*	1
	3321^*b*^	*Enterobacter xiangfangensis*	1

^*a*^ Species were identified with the ANI method.

^*b*^ Novel STs include ST3031, ST3243, ST3244, ST3321, and ST3323. ST78 and ST133 groups include their corresponding single-locus variants.

**Fig 3:**
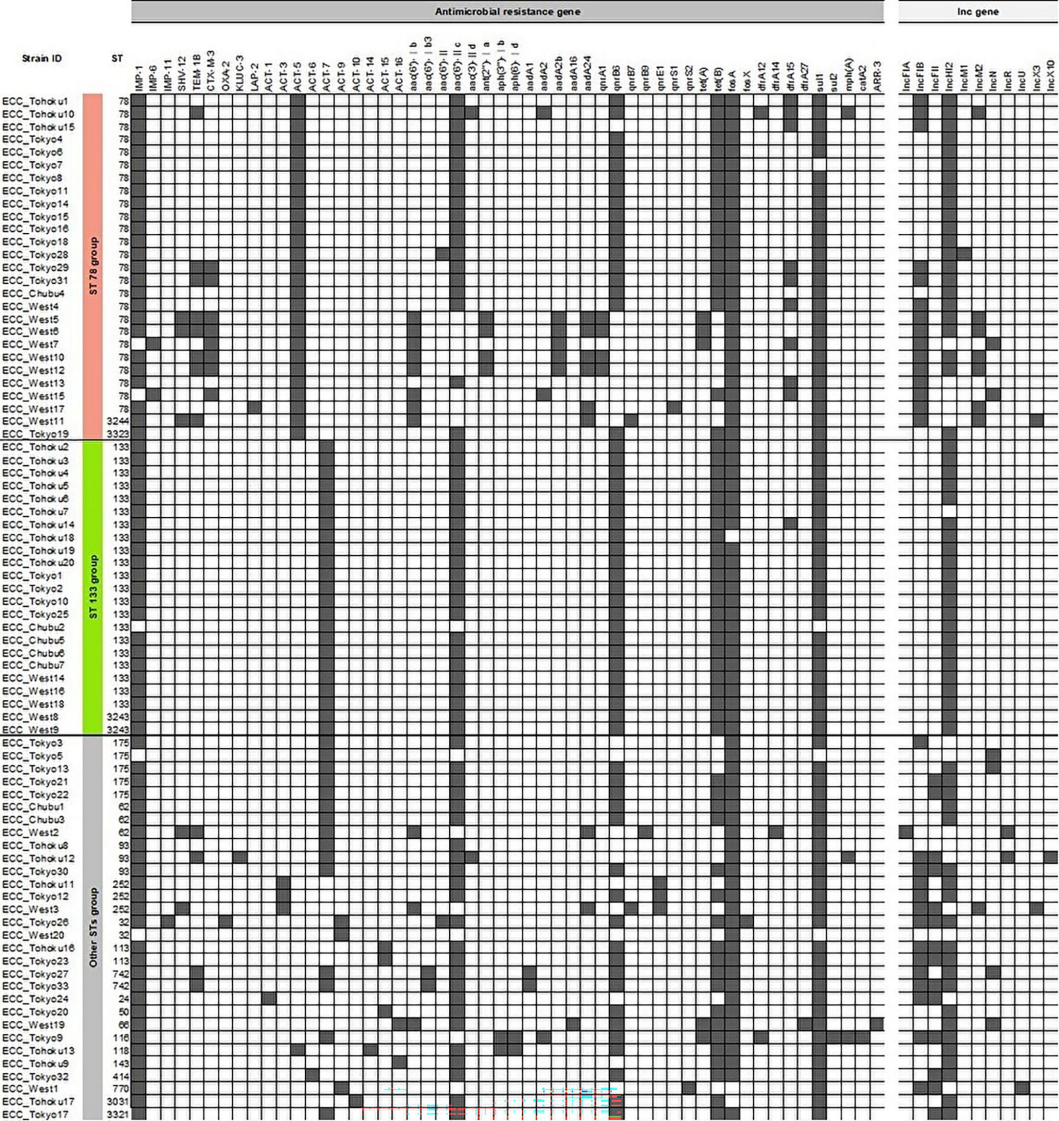
Distribution of antimicrobial resistance and plasmid Inc genes among 80 CPEC isolates. Each row represents a CPEC isolate, grouped and color-coded by ST: red for ST78 group, green for ST133 group, and gray for other STs group. Each column indicates the presence or absence of specific antimicrobial resistance genes (left block) or plasmid Inc genes (right block), as identified by WGS. Black squares indicate the presence of a gene (positive), and white squares indicate absence (negative). Genes are listed horizontally, and isolates are arranged vertically from the most to the least frequent ST.

WGS further revealed ST-specific resistance-gene and plasmid-replicon profiles (Fig. 3, Table 2). The *bla*_IMP-1_ was present in 93.8% of isolates (two Shikoku ST78 isolates carried *bla*_IMP-6_). Extended-spectrum beta-lactamase (ESBL) genes were common in the ST78 group (37.0%) and other STs group (16.7%), but absent in the ST133 group (adjusted *P* = 0.0024 vs ST78 group; *P* = 0.0184 vs other STs group); most ESBL-positive ST78 group isolates co-harbored IncHI2 and at least one additional plasmid replicon, although the specific combinations varied. ST78 group carried *bla*_ACT-5_, whereas ST133 group uniformly harbored *bla*_ACT-7_. Multi-replicon plasmids were significantly more frequent in the ST78 group (55.6%) and other STs group (66.7%) than in the ST133 group (0%; adjusted *P* < 0.0003); other STs group also possessed unique Inc types (IncR, IncU, and IncX10).

**TABLE 2:** Resistance genes and plasmid profiles in CPEC isolates by sequence type^*c*^^*,^d^*^

Resistance gene	Sequence type (s), *n* (%)		
Inc gene	ST78 group (*N* = 27)	ST133 group (*N* = 23)	Others STs group (*N* = 30)
IMP prevalence	27 (100)	22 (95.7)	28 (93.3)
*bla*_IMP-1_	25 (92.6)	22 (95.7)	28 (93.3)
*bla*_IMP-6_	2 (7.4)	0 (0.0)	0 (0.0)
*bla*_IMP-11_	0 (0.0)	0 (0.0)	1 (3.3)
ESBL prevalence^*a*^	10 (37.0)	0 (0.0)	5 (16.7)
*bla*_SHV-12_	3 (11.1)	0 (0.0)	2 (6.7)
*bla*_TEM-1B_	8 (29.6)	0 (0.0)	2 (6.7)
*bla*_CTX-M-3_	8 (29.6)	0 (0.0)	0 (0.0)
Inc gene prevalence	27 (100)	22 (95.7)	29 (96.7)
IncHI2	22 (81.5)	22 (95.7)	21 (70.0)
IncFIB	15 (55.6)	0 (0.0)	14 (46.7)
IncFII	0 (0.0)	0 (0.0)	14 (46.7)
IncM2	7 (25.9)	0 (0.0)	1 (3.3)
IncN	2 (7.4)	0 (0.0)	4 (13.3)
Multi-Inc carriage^*b*^	15 (55.6)	0 (0.0)	20 (66.7)

^*a*^ ESBL prevalence: Fisher’s exact test with Bonferroni correction. Adjusted *P* values—ST78 group vs ST133 group: 0.0024; ST133 group vs other STs group: 0.0184; ST78 group vs other STs group: 0.3939. Significant differences were found between ST78 and ST133 groups and between ST133 and other ST groups.

^*b*^ Multi-Inc carriage: adjusted *P* values—ST78 group vs ST133 group: <0.0003; ST133 group vs other STs group: <0.0003; ST78 group vs other STs group: 1.0000. Significant differences were found between ST78 and ST133 groups and between ST133 and other STs group.

^*c*^ Resistance genes or Inc groups detected in ≤2 isolates (including IncFIA, IncM1, IncR, IncU, IncX3, and IncX10) are omitted.

^*d*^ Inc, incompatibility; IMP, imipenemase; ST, sequence typing.

### Antimicrobial susceptibility of bla_IMP-1_ group-positive ECC isolates

To ensure standardized susceptibility testing, these 196 CPEC isolates were subsequently re-evaluated using the broth microdilution (BMD) method, and antimicrobial susceptibility was interpreted according to Clinical and Laboratory Standards Institute (CLSI) M100 breakpoints (19). Despite harboring carbapenemase genes, a large proportion remained susceptible to carbapenems, with 84.7% susceptible to imipenem and 83.7% to meropenem (Table 3). Among the 196 isolates, 153 (78.1%) were classified as susceptible to both imipenem and meropenem by the BMD method. Notably, 9 of these 153 isolates (5.9%) exhibited meropenem MIC ≤0.125 µg/mL (data not shown).

**TABLE 3:** Antimicrobial susceptibility profiles of *bla*_IMP-1_ group positive CPEC isolates

Antimicrobial agent	Susceptible isolates, *n* (%)			
	All isolates (*n* = 196)	ST78 group isolates (*n* = 25)^a^	ST133 group isolates (*n* = 22)^a^	*P*-value^b^
Imipenem	166 (84.7)	20 (80.0)	18 (81.8)	1
Meropenem	164 (83.7)	19 (76.0)	19 (86.4)	1
Piperacillin/tazobactam	105 (53.6)	11 (44.0)	9 (40.9)	1
Cefmetazole	1 (0.5)	0 (0.0)	0 (0.0)	1
Cefotaxime	1 (0.5)	1 (4.0)	0 (0.0)	1
Ceftazidime	1 (0.5)	1 (4.0)	0 (0.0)	1
Cefepime	25 (12.8)	1 (4.0)	4 (18.2)	0.170
Ceftolozane/tazobactam	2 (1.0)	1 (4.0)	0 (0.0)	1
Aztreonam	84 (42.9)	5 (20.0)	8 (36.4)	0.328
Colistin	187 (95.4)	25 (100.0)	21 (95.5)	1
Gentamicin	159 (81.1)	21 (84.0)	22 (100.0)	0.112
Amikacin	195 (99.5)	24 (96.0)	22 (100.0)	1
Tigecycline	173 (88.3)	18 (72.0)	18 (81.8)	0.505
Levofloxacin	87 (44.4)	0 (0.0)	15 (68.2)	<0.0001
Ciprofloxacin	83 (42.3)	0 (0.0)	14 (63.6)	<0.0001
Sulfamethoxazole/trimethoprim	108 (55.1)	12 (48.0)	17 (77.3)	0.070
Cefiderocol	195 (99.5)	25 (100.0)	22 (100.0)	1

^a^ ST78 group (*n* = 25) and ST133 group (*n* = 22) include only isolates in which *bla*_IMP-1_ was confirmed by WGS.

^b^ *P*-values were calculated using Fisher’s exact test comparing ST78 and ST133 groups. Statistical significance was defined as *P* < 0.05.

Regarding susceptibility to non-carbapenem agents, most isolates exhibited high susceptibility to colistin, amikacin, and cefiderocol. Subgroup analyses were conducted for CPEC isolates in which *bla*_IMP-1_ was confirmed by WGS, focusing on the ST78 group (*n* = 25) and ST133 group (*n* = 22). Both groups were uniformly susceptible to these agents. However, ST133 group isolates showed significantly higher susceptibility to fluoroquinolones than ST78 group (levofloxacin: 68.2% vs 0%; ciprofloxacin: 63.6% vs 0%; *P* < 0.0001). No statistically significant differences were observed in susceptibility to trimethoprim–sulfamethoxazole (77.3% vs 48.0%) or tigecycline (81.8% vs 72.0%) between ST133 and ST78 isolates. Among the 196 isolates, only one strain was resistant to cefiderocol.

### Clinical and epidemiological characteristics of ECC harboring bla_IMP-1_ group isolates and associated healthcare facilities

ECC isolates harboring the *bla*_IMP-1_ group were detected in 79.3% (46/58) of surveyed facilities, with 73.9% (34/46) of these being small hospitals with <200 beds. The average bed count among *bla*_IMP-1_ group-positive facilities was 174.6 (95% CI: 135.3–214.0), with a median of 145 beds. When stratified by hospital size (<200 vs ≥200 beds), the proportion of *bla*_IMP-1_ group-positive isolates among screened ECC was similarly high (90.5% vs 94.8%) and not significantly different (*P* = 1.00, Fisher’s exact test; see Table 4).

**TABLE 4:** Proportion of CPEC isolates harboring the *bla*_IMP-1_ group by facility size

Facility size (beds)	No. of facilities, *n* (%)	*bla*_IMP-1_ group among screened ECC isolates, % (*n*/*N*)^*a*^	Major outlier hospitals (isolates, *n*)
<200 beds	34 (73.9)	90.5 (86/95)	Hospital No.14 (9), No.37 (28)
≥200 beds	12 (26.1)	94.8 (110/116)	Hospital No.7 (88)

^*a*^ *N* indicates the number of screened ECC isolates that met the Japanese CRE criteria.

Hospital no. 7 alone accounted for 88 of the 196 CPEC isolates (44.9%). Using the interquartile range (IQR) method, three high-burden hospitals (nos. 7, 14, and 37) were identified as outliers and together contributed 63.8% (125/196) of all *bla*_IMP-1_ group-positive isolates. Hospitals nos. 14 and 37 were small (<200 beds), indicating that high detection of CPEC isolates was not limited to large institutions (≥200 beds). In contrast, the remaining 43 facilities reported far fewer cases, with a mean of 1.65 isolates per facility (95% CI, 1.35–1.95) over the 8-year surveillance period.

### Regional distribution of major STs among CPEC isolates

Both strain- and facility-level frequencies of ST78 and ST133 groups were similar across all regions (Table 5), with no significant differences after Bonferroni correction—indicating a uniformly distributed high-risk clone landscape. When limiting the analysis to regions with ≥10 sequenced isolates (Tohoku, Kanto [Tokyo], Kyushu) to ensure adequate power, the ST133 group was significantly more frequent in Tohoku than in Tokyo at the strain level (*P* = 0.0119), whereas no significant differences were observed at the facility level.

**TABLE 5:** Regional distribution of sequence types among 80 CPEC isolates analyzed by WGS^*c*^

Region	ST78 group		ST133 group		Other STs group	
	Isolates, *n* (%)	Facilities, *n* (%)	Isolates, *n* (%)	Facilities, *n* (%)	Isolates, *n* (%)	Facilities, *n* (%)
Tohoku	3 (15.0)	3 (30.0)	10 (50.0)^*a*^	4 (40.0)	7 (35.0)	6 (60.0)
Kanto (Tokyo)	13 (39.4)	8 (42.1)	4 (12.1)	4 (21.1)	16 (48.5)	12 (63.2)
Chubu	1 (14.3)	1 (20.0)	4 (57.1)	4 (80.0)	2 (28.6)	2 (40.0)
Chugoku	1 (25)	1 (50.0)	2 (50.0)	1 (50.0)	1 (25.0)	1 (50.0)
Kyushu	7 (53.8)	2 (28.6)	3 (23.1)	2 (28.6)	3 (23.1)	3 (42.9)
Shikoku	2 (66.7)^*b*^	2 (66.7)	0 (0.0)	0 (0.0)	1 (33.3)	1 (33.3)

^*a*^ *P* = 0.0119 (Bonferroni‐corrected) for ST133 group difference between Tohoku and Tokyo (regions with ≥10 isolates).

^*b*^ Two Shikoku ST78 isolates carried *bla*_IMP-6_.

^*c*^ Facilities may appear more than once if they yielded multiple STs.

### Genomic surveillance and transmission dynamics of high-risk CPEC clones in local and regional healthcare settings

**Fig 4:**
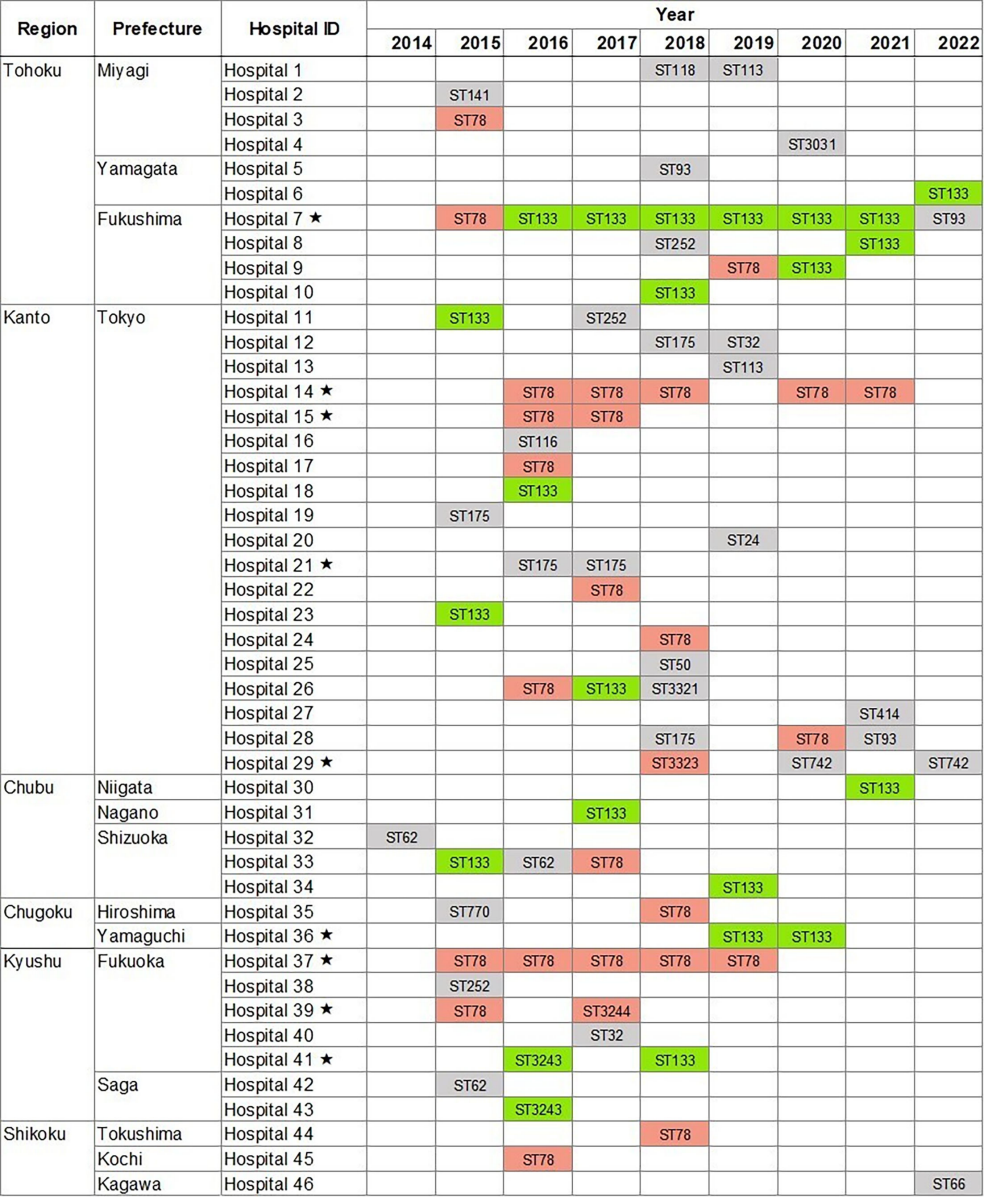
Temporal and regional trends of representative *bla*_IMP-1_ group-positive ECC isolates in Japan, 2014–2022. Each row represents a hospital, and each column a calendar year (2014–2022). Colored cells indicate the ST of the first CPEC isolate collected in that hospital and year; blood culture isolates were prioritized when available. STs are color coded by group: orange for ST78 group, green for ST133 group, and gray for other ST groups. A black star (⋆) marks facilities where the same ST was detected in ≥2 different years, suggesting possible clonal persistence. ST, sequence type; Inc, incompatibility.

Recurrent detection of the same ST across multiple years occurred in 9 of the 46 surveyed facilities (19.6%; Fig. 4), involving ST78 (four facilities), ST133 (three facilities), and other ST groups, such as ST175 and ST742 (two facilities). The recurrence proportion did not differ significantly between ST78 and ST133 groups (*P* = 1.00, Fisher’s exact test).

**Fig 5:**
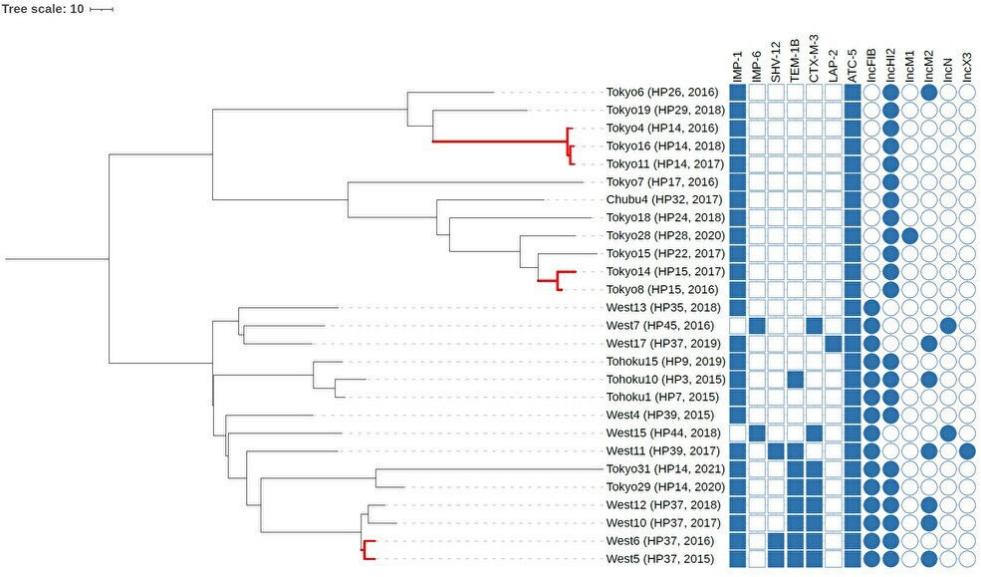
cgMLST allele-based phylogeny of ST78 group CPEC isolates. A neighbor-joining tree was constructed using allele distance from 3,992 cgMLST loci specific to the ST78 group. Strains connected by red branches differ by ≤10 alleles, indicating potential clonal dissemination. Labels include strain ID, hospital ID (in parentheses), and year of isolation. All ≤10-allele clusters were confined to individual hospitals. Columns on the right show the presence of β-lactamase genes and plasmid replicon types (Inc groups). Darker blue squares indicate gene presence. Strain IDs are shown without the “ECC_” prefix for simplicity in the figure.

**Fig 6:**
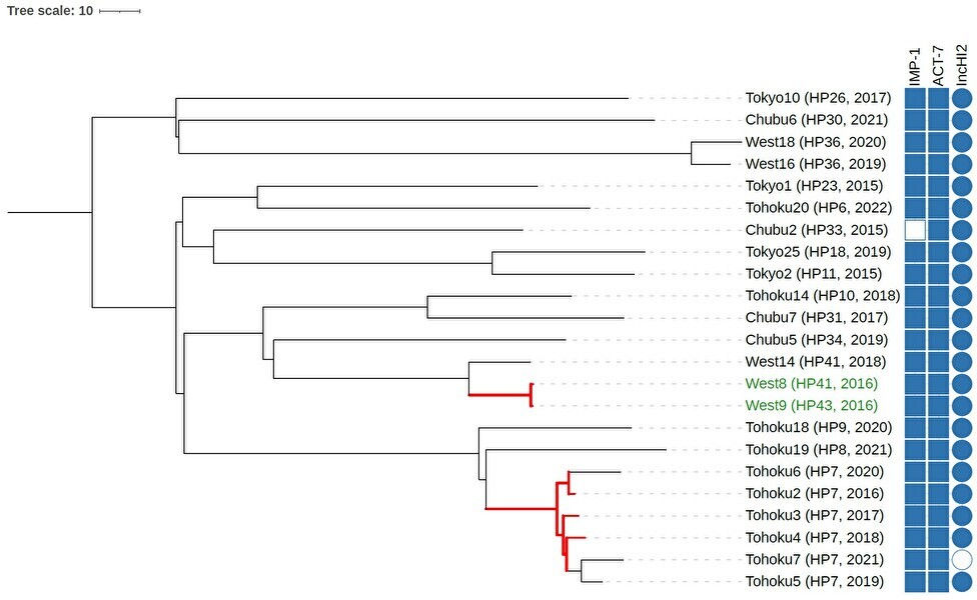
cgMLST allele-based phylogeny of ST133 group CPEC isolates. A neighbor-joining tree based on cgMLST data comprising 4,480 loci specific to ST133 group. Red branches indicate isolate pairs with ≤10 allelic differences. Strain IDs ECC_West8 and ECC_West9 (ST3243), shown in dark green, are genetically closely related despite being isolated in the same year from different hospitals located in adjacent prefectures. Columns indicate strain ID, β-lactamase genes, plasmid incompatibility (Inc) types, facility ID, and year of isolation. For simplicity, strain IDs in the figure are shown without the “ECC_” prefix. Inc, incompatibility group; ST, sequence type.

Core genome multi-locus sequence typing (cgMLST) allele-based analysis clarified potential transmission patterns (Fig. 5 and 6). Allelic differences between isolates are detailed in Tables 6 and 7. Among the ST78 group-positive facilities, genetically related strains (≤10 allele differences in cgMLST) were repeatedly identified over time in three hospitals (nos. 14, 15, and 37), indicating possible intra-facility transmission. Some facilities also showed replacement of strains by genetically distinct lineages, consistent with clonal turnover. In contrast, at hospital no. 7 (Fukushima), ST133 isolates remained genetically related from 2016 through 2018, and even after 2019, strains with relatively small allelic differences were detected. In Fukuoka (hospital No. 41), one ST133 group (ST3243) isolate differed by ≤10 alleles from an ST133 group (ST3243) isolate in neighboring Saga (hospital no. 43). Although most ST categories harbored *bla*_IMP-1_, notable differences in plasmid profiles, ESBL gene carriage, and phylogenetic structure indicate distinct evolutionary and transmission dynamics.

**TABLE 6:** Allelic differences between 27 CPEC ST78 group isolates^*a*^^,^*^b^*^,^^*c*^

ECC ST78	Chubu4	Tokyo8	Tohoku1	Tohoku10	Tohoku15	Tokyo11	Tokyo14	Tokyo15	Tokyo16	Tokyo18	Tokyo19	Tokyo28	Tokyo29	Tokyo31	Tokyo4	Tokyo6	Tokyo7	West10	West11	West12	West13	West15	West17	West4	West5	West6	West7
Chubu4	**0**	88	262	273	265	269	93	100	270	105	253	100	287	357	268	244	169	296	262	292	259	263	249	247	288	288	256
Tokyo8	88	**0**	270	278	269	282	**9**	32	283	97	266	43	293	362	281	253	175	298	273	292	271	274	260	258	288	287	267
Tohoku1	262	270	**0**	16	25	277	271	284	276	280	257	270	131	212	276	242	283	123	101	118	103	103	92	91	115	115	97
Tohoku10	273	278	16	**0**	31	285	284	293	284	288	267	282	138	219	284	253	291	131	109	127	111	111	99	99	124	124	106
Tohoku15	265	269	25	31	**0**	278	275	284	277	280	258	275	132	211	278	243	283	121	99	117	101	101	91	90	114	114	96
Tokyo11	269	282	277	285	278	**0**	286	294	**3**	295	94	278	297	369	**4**	98	287	300	278	298	279	280	267	267	293	294	271
Tokyo14	93	**9**	271	284	275	286	**0**	39	287	103	271	46	300	366	286	258	184	303	277	298	276	280	265	265	294	293	272
Tokyo15	100	32	284	293	284	294	39	**0**	295	109	278	49	307	376	293	265	192	317	286	312	283	286	272	271	308	307	277
Tokyo16	270	283	276	284	277	**3**	287	295	**0**	296	93	279	298	370	**5**	100	286	299	277	297	278	279	266	266	292	293	270
Tokyo18	105	97	280	288	280	295	103	109	296	**0**	278	110	306	375	293	264	190	308	285	303	282	288	272	272	299	298	278
Tokyo19	253	266	257	267	258	94	271	278	93	278	**0**	263	277	353	92	83	271	280	256	278	258	261	247	248	273	274	252
Tokyo28	100	43	270	282	275	278	46	49	279	110	263	**0**	300	364	278	255	189	307	277	303	274	278	261	263	298	298	271
Tokyo29	287	293	131	138	132	297	300	307	298	306	277	300	**0**	101	296	260	304	110	97	106	126	115	115	105	101	101	120
Tokyo31	357	362	212	219	211	369	366	376	370	375	353	364	101	**0**	367	335	373	190	177	186	203	194	193	186	180	180	198
Tokyo4	268	281	276	284	278	**4**	286	293	**5**	293	92	278	296	367	**0**	97	286	299	278	298	278	279	266	266	293	294	270
Tokyo6	244	253	242	253	243	98	258	265	100	264	83	255	260	335	97	**0**	259	264	247	262	245	249	237	236	258	260	241
Tokyo7	169	175	283	291	283	287	184	192	286	190	271	189	304	373	286	259	**0**	307	277	304	273	276	265	264	299	300	270
West10	296	298	123	131	121	300	303	317	299	308	280	307	110	190	299	264	307	**0**	96	18	120	112	108	103	21	21	115
West11	262	273	101	109	99	278	277	286	277	285	256	277	97	177	278	247	277	96	**0**	92	98	88	88	79	87	86	93
West12	292	292	118	127	117	298	298	312	297	303	278	303	106	186	298	262	304	18	92	**0**	117	108	103	97	13	13	112
West13	259	271	103	111	101	279	276	283	278	282	258	274	126	203	278	245	273	120	98	117	**0**	100	69	91	112	113	73
West15	263	274	103	111	101	280	280	286	279	288	261	278	115	194	279	249	276	112	88	108	100	**0**	89	80	104	103	95
West17	249	260	92	99	91	267	265	272	266	272	247	261	115	193	266	237	265	108	88	103	69	89	**0**	80	99	99	59
West4	247	258	91	99	90	267	265	271	266	272	248	263	105	186	266	236	264	103	79	97	91	80	80	**0**	93	93	84
West5	288	288	115	124	114	293	294	308	292	299	273	298	101	180	293	258	299	21	87	13	112	104	99	93	**0**	**8**	107
West6	288	287	115	124	114	294	293	307	293	298	274	298	101	180	294	260	300	21	86	13	113	103	99	93	**8**	**0**	108
West7	256	267	97	106	96	271	272	277	270	278	252	271	120	198	270	241	270	115	93	112	73	95	59	84	107	108	**0**

^*a*^ Allelic differences of 10 or fewer—defined as closely related—are highlighted in bold.

^*b*^ Each cell shows the number of allelic differences between isolate pairs, calculated using the cgMLST scheme in Ridom SeqSphere+.

^*c*^ For simplicity, strain IDs in the figure are shown without the “ECC_” prefix.

**TABLE 7:** Allelic differences between 23 CPEC ST133 group isolates^*a*^^,^*^b^*^,^^*c*^

ECC ST133	West9	Chubu2	Chubu5	Chubu6	Chubu7	Tohoku14	Tohoku18	Tohoku19	Tohoku2	Tohoku20	Tohoku3	Tohoku4	Tohoku5	Tohoku6	Tohoku7	Tokyo1	Tokyo10	Tokyo2	Tokyo25	West14	West16	West18	West8
West9	**0**	160	126	214	141	129	180	187	167	171	165	168	169	177	175	157	217	179	181	28	246	250	**1**
Chubu2	160	**0**	168	221	179	164	177	188	167	170	166	171	170	176	175	158	220	162	167	160	244	244	160
Chubu5	126	168	**0**	231	150	135	186	193	174	175	174	177	178	184	183	164	225	186	184	120	252	256	126
Chubu6	214	221	231	**0**	246	236	250	252	238	239	235	241	239	248	247	222	208	239	247	214	230	232	215
Chubu7	141	179	150	246	**0**	76	197	207	187	196	187	190	190	195	194	182	242	198	203	140	260	261	141
Tohoku14	129	164	135	236	76	**0**	187	196	175	186	173	176	177	182	181	170	228	190	194	129	256	258	129
Tohoku18	180	177	186	250	197	187	**0**	76	57	191	57	58	62	64	65	183	242	202	205	178	263	264	180
Tohoku19	187	188	193	252	207	196	76	**0**	60	200	60	64	66	70	71	190	249	213	214	186	268	270	187
Tohoku2	167	167	174	238	187	175	57	60	**0**	183	**9**	**10**	14	13	18	171	230	196	198	167	251	253	167
Tohoku20	171	170	175	239	196	186	191	200	183	**0**	183	187	186	193	191	137	232	188	190	173	256	259	172
Tohoku3	165	166	174	235	187	173	57	60	**9**	183	**0**	**8**	13	20	17	171	227	193	197	165	248	251	165
Tohoku4	168	171	177	241	190	176	58	64	**10**	187	**8**	**0**	12	20	17	175	231	198	200	169	252	256	168
Tohoku5	169	170	178	239	190	177	62	66	14	186	13	12	**0**	24	14	174	231	198	201	169	253	254	169
Tohoku6	177	176	184	248	195	182	64	70	13	193	20	20	24	**0**	30	180	237	204	207	177	260	261	177
Tohoku7	175	175	183	247	194	181	65	71	18	191	17	17	14	30	**0**	181	238	204	202	175	259	263	175
Tokyo1	157	158	164	222	182	170	183	190	171	137	171	175	174	180	181	**0**	222	181	181	155	243	246	158
Tokyo10	217	220	225	208	242	228	242	249	230	232	227	231	231	237	238	222	**0**	235	239	217	225	228	217
Tokyo2	179	162	186	239	198	190	202	213	196	188	193	198	198	204	204	181	235	**0**	66	178	263	266	179
Tokyo25	181	167	184	247	203	194	205	214	198	190	197	200	201	207	202	181	239	66	**0**	179	264	269	181
West14	28	160	120	214	140	129	178	186	167	173	165	169	169	177	175	155	217	178	179	**0**	246	249	28
West16	246	244	252	230	260	256	263	268	251	256	248	252	253	260	259	243	225	263	264	246	**0**	20	245
West18	250	244	256	232	261	258	264	270	253	259	251	256	254	261	263	246	228	266	269	249	20	**0**	248
West8	**1**	160	126	215	141	129	180	187	167	172	165	168	169	177	175	158	217	179	181	28	245	248	**0**

^*a*^ Allelic differences of 10 or fewer—defined as closely related—are highlighted in bold.

^*b*^ Each cell shows the number of allelic differences between isolate pairs, calculated using the cgMLST scheme in Ridom SeqSphere+.

^*c*^ For simplicity, strain IDs in the figure are shown without the “ECC_” prefix.

## DISCUSSION

In this study, 211 ECC isolates screened using an automated system based on Japan’s national CRE criteria were subjected to PCR testing, which detected *bla*_IMP-1_ group in 196 isolates. WGS of representative isolates revealed the predominance of high-risk clonal groups, with evidence suggesting clonal persistence in some hospitals.

WGS of representative isolates identified two dominant international high-risk clones ST78 (*E. hormaechei*) and ST133 (*Enterobacter xiangfangensis*) (20). ST78 is a globally disseminated lineage associated with multidrug resistance and hospital adaptation (4, 21) and has caused IMP-producing ECC outbreaks in Japan (12, 22). ST133 is rarely reported worldwide or domestically despite prior descriptions of siderophore-associated hypervirulence in infection models (13, 23, 24). Despite limited prior reports, ST133 emerged as a prominent lineage in our study, suggesting that it may be more widespread than previously recognized. Its recurrent detection over multiple years within the same hospitals suggests clonal persistence and gradual, previously unrecognized dissemination. These findings underscore the epidemiological relevance of both ST78 and ST133, highlighting the need for geographically broader genomic surveillance to monitor high-risk CPEC lineages.

The enrichment of ESBL genes and frequent co-carriage of multiple plasmid replicons in the ST78 group suggests that this clone may have acquired additional resistance mechanisms that enhance its ability to persist or disseminate under antimicrobial pressure in hospital settings (21). This contrast in genetic profiles highlights potential differences in selective pressures or evolutionary pathways between the two clones. In addition to ESBL genes, ST78 group strains uniformly exhibited resistance to fluoroquinolone antibiotics. Other mechanisms—such as mutations in the quinolone resistance-determining regions of gyrA and parC, plasmid-mediated quinolone resistance genes, and overexpression of efflux pumps—may also contribute to this phenotype and warrant further investigation (25).

Despite these differences, all isolates—regardless of ST—remained highly susceptible to amikacin, colistin, tigecycline, and cefiderocol. Our cefiderocol results align with national data reporting ≈99.2% IMP-type *Enterobacterales* susceptibility (26). Cefiderocol resistance may be associated with cirA mutations (27), although further investigation into these mechanisms is warranted.

Current Infectious Diseases Society of America (IDSA) guidelines recommend ceftazidime–avibactam plus aztreonam or cefiderocol for MBL producers (28). This emerging resistance, albeit infrequent, highlights the necessity for ongoing molecular surveillance of cefiderocol susceptibility—so that treatment can adapt promptly to shifting resistance landscapes.

In Japan, the healthcare system operates as a hierarchical network encompassing advanced acute-care centers (e.g., university and large specialist hospitals) and numerous small- to medium-sized local facilities (29, 30). Patients are frequently transferred between institutions according to clinical need and disease severity, facilitating appropriate care while also increasing the potential for interfacility transmission of antimicrobial-resistant organisms, particularly CRE. Moreover, while active CRE infections are notifiable and subject to public health oversight, colonization is not reportable, and policies for managing asymptomatic carriers vary by institution. Although official recommendations for CRE infection control, as well as national guidelines for antimicrobial stewardship, exist (31, 32), their implementation may vary considerably between institutions, possibly due to differences in infection control resources—particularly in smaller or non-tertiary hospitals. A large proportion of medical institutions are classified as small hospitals with fewer than 200 beds (33), and in our study, the majority of surveyed facilities also fell into this category. Although small hospitals often report fewer CPEC cases, their potentially limited capacity for infection control may increase their vulnerability as reservoirs for drug-resistant organisms (34). In practice, one contributing factor to the high proportion of small hospitals in our study may be the outsourcing of microbiological testing due to limited in-house resources. Although previous studies have described sporadic, low‐frequency dissemination of long‐term, clone‐related CRE/CPE in large tertiary centers (35, 36), our phylogenetic analyses extend this observation to small hospitals, showing a similar pattern of infrequent yet persistent clone spread. These findings highlight the importance of genomic analysis as a complementary tool in surveillance efforts to distinguish clonal persistence from repeated introductions (37). To suppress the spread of high‐risk clones in a decentralized healthcare network, infection‐control strategies must be tailored not only to hospital size but also to each institution’s specific risk profile (38). Combined analyses of genomic and epidemiological data have already demonstrated their utility in elucidating complex transmission pathways and enabling timely, targeted interventions during nosocomial outbreaks (17, 39). In resource‐constrained small hospitals, leveraging external guidance to implement standardized monitoring and education programs may enhance the effectiveness of infection‐control measures (40).

Detecting IMP-1-producing ECC remains complex, particularly due to occasional discordance between genotypic resistance and phenotypic susceptibility, meaning that some isolates may appear susceptible to carbapenems despite harboring resistance genes (12, 13). Although automated susceptibility systems may overestimate carbapenem resistance in some cases, similar discrepancies have been previously reported (41, 42). In this study, several *bla*_IMP-1_ group-positive isolates exhibited low meropenem MICs (≤0.125 µg/mL) as determined by BMD, highlighting the limitations of phenotypic screening alone, as even low MICs may not exclude carbapenemase production (43). The April 2025 revision of Japan’s CRE definition now permits confirmation by either a meropenem MIC ≥2 µg/mL or a meropenem disk inhibition zone ≤22 mm, or alternatively by molecular/immunochromatographic methods (44). However, practical criteria for selecting isolates for confirmatory testing remain unclear. In regions where IMP-1-producing ECC is known to be prevalent, molecular confirmation may be warranted even for isolates with low meropenem MICs. This underscores the importance of understanding local epidemiology to guide appropriate diagnostic strategies.

This study had some limitations. First, representative isolates were selected to encompass regional and temporal diversity; however, rare STs and isolates from the Kinki region centered on Osaka—where *bla*_IMP-6_ is prevalent—were not included; thus, our findings may not be fully generalizable to the entire country (45). Second, the absence of patient‐level clinical data, including movement between wards and clinical outcomes, prevented us from distinguishing infection from colonization and also constrained our ability to reconstruct precise transmission pathways. Third, our PCR‐based screening was restricted to major carbapenemase genes and may have overlooked uncommon variants or non‐enzymatic resistance mechanisms such as porin loss and AmpC overexpression (46); draft genome assembly gaps may have also obscured certain genetic elements such as antimicrobial resistance genes, mobile genetic elements, or plasmid replication genes (47). Finally, the overrepresentation of isolates from one high-burden facility and variable participation rates across hospitals—some of which contributed more isolates due to higher case numbers—may have introduced sampling bias and influenced the observed distribution of STs. Although we included all non-duplicate ECC isolates meeting the screening criteria from surveyed regions, our findings may not be fully generalizable to the nationwide CPEC epidemiology in Japan. Despite these limitations, this study represents a comprehensive genomic analysis of *bla*_IMP-1_-producing ECC in Japan. Future research should expand geographic coverage, incorporate clinical and epidemiological data, and investigate plasmid transmission to guide infection control.

In conclusion, these findings highlight the potential for the silent dissemination of high-risk CPEC clones such as ST133 in decentralized settings, emphasizing the importance of integrating molecular diagnostics and genomic surveillance into CRE control efforts.

## MATERIALS AND METHODS

### Study design

This retrospective study analyzed archived clinical isolates of ECC strains collected from a range of healthcare facilities across different regions in Japan to ensure geographic and institutional diversity. Ethical approval was obtained from the Ethics Committee of the Tohoku University Graduate School of Medicine (2024-1-284).

### Bacterial isolates

Between September 2014 and December 2022, clinical specimens submitted for diagnostic testing from various regions in Japan were processed at the Biomedical Laboratories R&D Center (Kawagoe, Saitama, Japan). Initial antimicrobial susceptibility screening was performed using the Microscan WalkAway system (Beckman Coulter, Brea, CA, USA) with Microscan Neg panels (Neg Combo EN 4J and Neg MIC EN 2J), and bacterial suspensions were prepared using the prompt inoculation method (48). All confirmed isolates were preserved at –40°C, and before further analysis, the purity of these frozen stocks was verified by species identification using the VITEK MS system (Sysmex BioMérieux, France). CRE were defined in accordance with the Infectious Disease Control Law of Japan as isolates meeting one of the following criteria: meropenem MIC ≥2 µg/mL, or both imipenem MIC ≥2 µg/mL and cefmetazole MIC ≥64 µg/mL (10). To avoid duplicate sampling, only one isolate per patient was included. Patient identity was linked to each specimen via anonymized identifiers, allowing accurate tracking across multiple submissions. When multiple specimens from the same individual yielded ECC isolates, the earliest specimen was selected; blood culture isolates were prioritized when available. If isolates were obtained from the same individual at different time points during the study period, only the first episode was considered. Isolates were collected from six geographically diverse regions of Japan (Tohoku, Kanto, Chubu, Chugoku, Shikoku, and Kyushu). We focused on typical local and regional hospitals, excluding university hospitals, psychiatric institutions, and long-term care facilities. Facility information was obtained from the Medical Information Net (Navii) database managed by the Ministry of Health, Labor, and Welfare (in Japanese; https://www.iryou.teikyouseido.mhlw.go.jp/znk-web/juminkanja/S2300/initialize). Medical facilities were classified into two groups based on bed capacity—<200 and ≥200 beds—in accordance with domestic administrative criteria (34).

### Microbiological and antimicrobial susceptibility testing

Initial screening was performed using the Microscan WalkAway system. For confirmatory testing, MICs were determined via the CLSI-standard BMD method (19, 49), using a customized 96-well frozen plate (Eiken, Tokyo, Japan) and following the manufacturer’s instructions. Bacterial suspensions were adjusted to a 0.5 McFarland turbidity in sterile saline and subsequently streaked onto Mueller–Hinton agar plates. *Escherichia coli* ATCC 25922 served as the quality control strain for all assays. The customized Eiken frozen plate included the following agents: imipenem, meropenem, piperacillin–tazobactam, cefmetazole, cefotaxime, ceftazidime, cefepime, ceftolozane–tazobactam, aztreonam, colistin, gentamicin, amikacin, tigecycline, levofloxacin, ciprofloxacin, trimethoprim–sulfamethoxazole, and cefiderocol. For cefiderocol testing, iron-depleted cation-adjusted Mueller–Hinton broth (ID-CAMHB) was used per CLSI recommendations (19). MICs were interpreted according to CLSI guidelines (19, 49), with Food and Drug Administration breakpoints applied for tigecycline (50).

### Identification of carbapenemase genes by PCR testing

PCR testing was performed on ECC isolates that showed reduced susceptibility to carbapenems during initial screening with the Microscan WalkAway system to detect carbapenemase genes. The testing was performed using the Cica Geneus Carbapenemase Genotype Detection Kit 2 (Kanto Chemical Co., Tokyo, Japan). This assay detects the following gene groups: *bla*_IMP-1_, *bla*_VIM_, *bla*_GES_, *bla*_KPC_, *bla*_NDM_, and *bla*_OXA-48_. Testing procedures followed the manufacturer’s protocol.

### WGS and data analysis

WGS was performed on *bla*_IMP-1_ group-positive isolates selected to ensure broad geographic, temporal, and institutional diversity; to capture potential long-term clonal persistence and track clone dynamics within each facility, we selected the first CPEC isolate recovered per facility in each calendar year for WGS, prioritizing blood culture isolates when available. Furthermore, genomic DNA was extracted using the QIAamp DNA Mini QIAcube Kit (QIAGEN), and libraries were prepared using the Nextera XT DNA Library Prep Kit (Illumina). Paired-end 2 × 300 bp reads. Reads were quality-trimmed and assembled *de novo* using SPAdes v3.13.0 (51). Species within the CPEC were assigned by ANI ≥95% via DFAST Taxonomy Check (52, 53), in accordance with a genome-based taxonomic framework that reclassifies several *Enterobacter* species as distinct species (54). MLST by PubMLST (https://pubmlst.org/) acquired resistance genes by ResFinder v4.6.0 with default thresholds (≥90% identity and ≥60% minimum length) (55, 56), *bla*_IMP_ variant confirmation via BLAST (NG_049172.1, NG_049220.1, and NG_049174.1; https://blast.ncbi.nlm.nih.gov/Blast.cgi), and plasmid replicons by PlasmidFinder v2.1 with default thresholds (≥95% identity and ≥60% coverage) (56, 57).

### Phylogenetic analysis based on core-genome MLST allele differences

A neighbor-joining tree was inferred from the core-genome alignment and cgMLST matrix generated in Ridom SeqSphere + v6.0.2 (Ridom GmbH, Münster, Germany; https://www.ridom.de/) (58), using *E. cloacae* AR_0050 (GenBank CP021896) and *E. hormaechei* C210017 (GenBank NZ_JAMHKI010000001) as seed reference genomes for ST78 and ST133 groups, respectively. The phylogenetic tree was visualized using iTOL v6 (https://itol.embl.de/). In accordance with previous studies, isolates exhibiting ≤10 allele differences in cgMLST were regarded as genetically related isolates (59, 60). Recurrent detection was defined as the isolation of the same ST from a single facility in multiple calendar years during the surveillance period (2014–2022). The proportion of such facilities was calculated as a percentage of all surveyed institutions.

### Statistical analysis

Outliers in isolate counts per facility were identified using the IQR method. Fisher’s exact test was used to assess differences in proportions, and pairwise comparisons were performed when ≥3 groups were analyzed. For multiple comparisons, Bonferroni correction was applied, and statistical significance after correction was defined as an adjusted *P*-value < 0.05. Analyses were performed using JMP Pro 16 (SAS Institute, Cary, NC, USA). All data were complete, without missing values. Statistical significance was defined as a *P*-value < 0.05.

## Data Availability

The results of this study were deposited in the NCBI database under BioProject number PRJDB18403. The draft genome sequences of the CPEC isolates were deposited in DDBJ and the NCBI.

## SUPPLEMENTAL MATERIAL

Supplemental tables aac.00953-25-s0001.xlsx Tables S1 to S3.

ASM does not own the copyrights to Supplemental Material that may be linked to, or accessed through, an article. The authors have granted ASM a non-exclusive, world-wide license to publish the Supplemental Material files. Please contact the corresponding author directly for reuse.
